# Effectiveness of Injectable Platelet-Rich Fibrin Therapy in Alopecia and Facial Rejuvenation: A Systematic Review

**DOI:** 10.7759/cureus.62198

**Published:** 2024-06-11

**Authors:** Sandip A Mohale, Pallav V Thakare, Sagar S Gaurkar, Gopikishan Bharadia, Sourya Acharya

**Affiliations:** 1 Medicine, Jawaharlal Nehru Medical College, Datta Meghe Institute of Higher Education and Research, Wardha, IND; 2 Internal Medicine, Jawaharlal Nehru Medical College, Datta Meghe Institute of Higher Education and Research, Wardha, IND; 3 Otolaryngology - Head and Neck Surgery, Jawaharlal Nehru Medical College, Datta Meghe Institute of Higher Education and Research, Wardha, IND; 4 Internal Medicine, Vivekanand Hospital, Latur, IND

**Keywords:** injectable therapy, regenerative dermatology, facial rejuvenation, alopecia, prf, platelet-rich fibrin

## Abstract

Platelet-rich fibrin (PRF) has gained attention in regenerative medicine for its potential to enhance tissue repair and regeneration. Its application in dermatology, particularly for treating alopecia and facilitating facial rejuvenation, is of significant interest but requires systematic evaluation. This review aims to systematically assess the effectiveness of injectable PRF therapy in treating alopecia and facial rejuvenation procedures. We searched PubMed, Scopus, and Web of Science for studies published up to 2023 that involved injectable PRF for alopecia and facial rejuvenation. Eligible studies included prospective cohorts, original studies, case series, and retrospective studies that reported clinical outcomes. Key outcomes were improved hair growth for alopecia and skin texture, elasticity, and appearance for facial rejuvenation. Seven studies met the inclusion criteria, encompassing 130 patients. For alopecia, three studies reported a noticeable improvement in hair density and growth. For facial rejuvenation, four studies demonstrated moderate to significant improvements in skin texture and elasticity and a reduction in facial wrinkles and lines, confirmed by both subjective assessments and objective measurements such as VISIA® skin analysis. Injectable PRF therapy shows promise in treating androgenetic alopecia and enhancing facial esthetics, indicating its potential as an effective treatment option in regenerative dermatology. However, further research involving larger sample sizes, control groups, and longer follow-ups is required to validate these findings and establish standardized treatment protocols.

## Introduction and background

Alopecia and aging-related facial skin changes are prevalent dermatological conditions that significantly affect individuals' quality of life. Innovative treatments, particularly those utilizing regenerative medicine techniques, have gained prominence in dermatological therapy due to their potential to improve tissue repair and regeneration [1]. Platelet-rich fibrin (PRF), a second-generation platelet concentrate, has been extensively studied. PRF is derived from autologous blood and, due to its rich content of growth factors and cytokines, is believed to promote healing and tissue regeneration [2,3]. PRF has been employed in various medical fields, such as dentistry, orthopedics, and cosmetic surgery, showing benefits in wound healing, bone regeneration, and esthetic improvements [4,5]. In dermatology, PRF is increasingly used for treating alopecia and facial rejuvenation. For alopecia, particularly androgenetic alopecia, PRF is hypothesized to stimulate hair follicles and improve hair density by enhancing vascularization and follicular cell proliferation [6,7].

Facial rejuvenation treatments with PRF aim to improve skin texture, elasticity, and overall appearance. The growth factors in PRF are known to stimulate collagen production, which is crucial in maintaining skin elasticity and strength, thereby potentially reducing wrinkles and improving skin texture [8,9]. Clinical trials and cohort studies have shown promising results in using PRF for facial esthetic improvements, including reducing the appearance of wrinkles and fine lines [10,11]. Despite these promising applications, the evidence is still emerging, and the mechanisms by which PRF exerts its effects still need to be fully understood. Comparative studies have highlighted the potential of PRF over other treatments, such as platelet-rich plasma (PRP), suggesting that PRF may offer a longer-term release of growth factors, which could be beneficial for prolonged tissue regeneration processes [12,13]. Current systematic reviews and meta-analyses are crucial for consolidating this diverse body of evidence, evaluating the efficacy of PRF, and establishing standardized treatment protocols. Such reviews can also identify gaps in the current research landscape, thereby guiding future studies [14]. With the increasing demand for minimally invasive treatments in cosmetic dermatology, the role of PRF could be pivotal if supported by robust clinical evidence [15].

## Review

Study design and protocol

This systematic review was meticulously designed by the PRISMA (Preferred Reporting Items for Systematic Reviews and Meta-Analyses) [16] guidelines to determine the efficacy of injectable PRF therapy in treating alopecia and facial rejuvenation. The objective was clearly defined: to analyze and synthesize existing research on the use of injectable PRF for improving clinical outcomes in dermatological applications.

Search strategy

**Figure 1:**
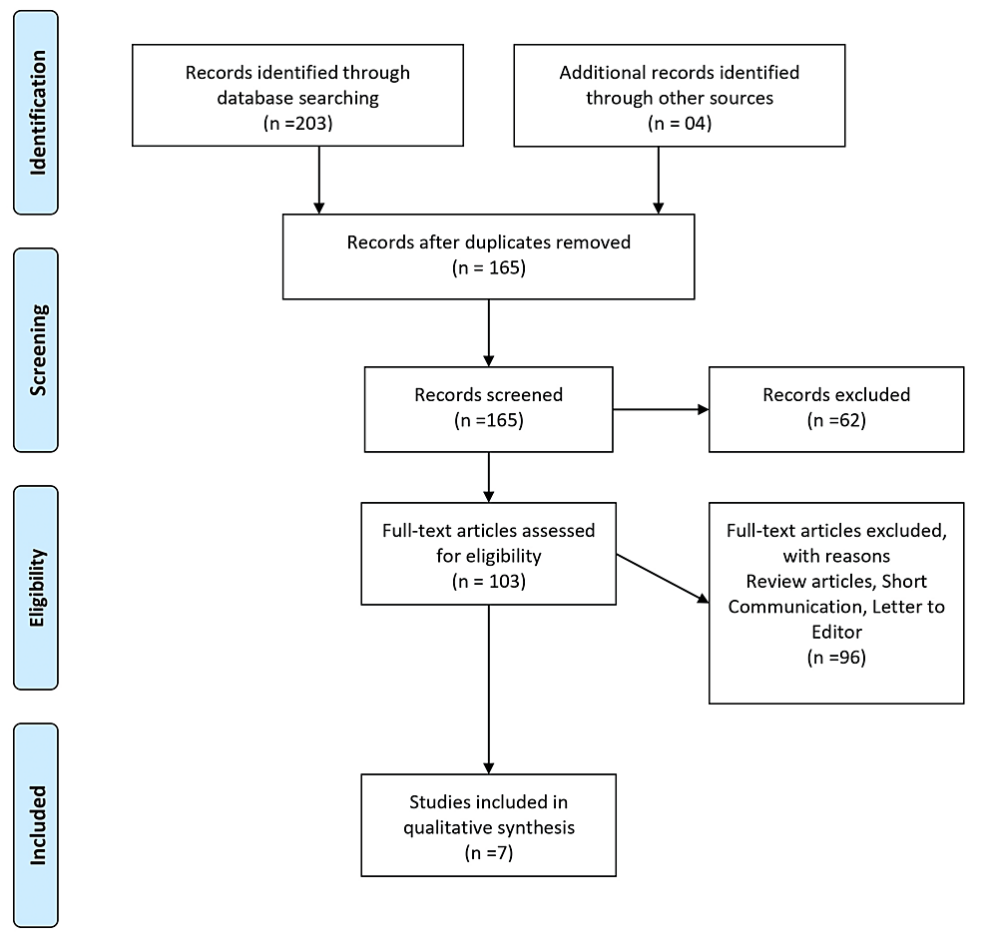
The selection process of articles used in this study. Adopted from the Preferred Reporting Items for Systematic Reviews and Meta-Analyses (PRISMA).

An exhaustive search was conducted across multiple databases, including PubMed, Scopus, and Web of Science, to compile relevant literature, targeting publications up to 2023. The search employed a combination of keywords such as "platelet-rich fibrin," "PRF," "injectable PRF," "alopecia," "hair loss treatment," "facial rejuvenation," and "dermatological applications." The aim was to capture all pertinent studies that provided clinical data on the outcomes of PRF treatments, focusing on those that involved intradermal injections of PRF. Figure 1 illustrates the selection procedure for the papers included in the present study, and Table 1 displays the characteristics of the articles used in this review.

**Table 1:** Characteristics of the included studies.

Author and Year	Study Type	Inclusion Criteria	Intervention	Study Group Characteristics	Study Outcome
Atsu N, et al. 2023 [17]	Prospective cohort	Adults who underwent facial skin rejuvenation and received intradermal injections were included.	The study groups utilized the patient's own whole blood to prepare either PRP or PRF for injection.	Total- 55, PRP- 23, PRF- 32. Mean age- 36.4 ± 8.9 years	The findings revealed no substantial difference between the two groups regarding the alterations in frontal and nasolabial parameters over time. Nevertheless, the PRF group demonstrated superior scores in canthal smoothness and wrinkle reduction, as evidenced by the p-values of 0.025 and 0.028. On the other hand, no difference was observed in canthal roughness and scaliness between the two groups, with p-values greater than 0.05 for both measures.
Vesala AM, et al. 2023 [11]	Original study	Patients with facial wrinkles were enrolled in the study.	The patients' whole blood was used to prepare the PRF for injection. Three i-PRF therapy sessions were provided at 4-week intervals using a Dermapen.	Total- 12. Mean age- 44 ± 13.6 years	Moderate improvements in skin texture, color, and elasticity (p = 0.02) and the filling of soft wrinkles (p = 0.001) were observed following three i-PRF sessions.
Bhoite KS, et al. 2022 [18]	Case series	Patients diagnosed with androgenetic alopecia received treatment with PRF injections.	Patients received PRF injections using their own whole blood.	15 patients	11 out of 15 patients exhibited a clinically significant improvement in hair growth.
Butt G, et al. 2020 [25]	Prospective study	Healthy individuals aged 18 or older with no prior facial surgery or nonsurgical treatments within 12 months, agreeing to avoid them during the study/treatment period, no active skin disease, no smoking, and no contraindications for aesthetic interventions.	The i-PRF treatment was administered using the PRF® PROCESS system technology. The study included 11 healthy female participants, who received monthly intradermal injections of i-PRF in three facial areas (malar regions, nasolabial folds, and upper lip skin) for a period of three months. The efficacy of the treatment was assessed using both objective skin analysis (VISIA®) and a subjective patient-reported outcome (FACE-Q) at the beginning and end of the study.	11 patients with age criteria- >18	At the end of the third month, it was observed that there were significant improvements in the appearance of skin surface spots and pores P < .05 for both and there was also a numerical improvement in other variables such as skin texture wrinkles ultraviolet spots porphyrins. additionally the face-q scales showed significant overall satisfaction with appearance including facial cheeks lower face jawline lips from baseline.
Shashank B, et al. 2021 [20]	Case series	Patients with a diagnosis of androgenetic alopecia were treated with injectable PRF.	PRF was prepared for injection using the whole blood of each patient, and injectable PRF was offered to them.	Total patients: 4	Injectable PRF was useful in treating androgenetic alopecia, rejuvenating undereye areas, temporary correction of facial skin folds, and healing difficult-to-treat wounds and ulcers.
Arora R, et al. 2019 [21]	Case series	Patients with a diagnosis of androgenetic alopecia were treated with injectable PRF.	PRF was prepared for injection using the whole blood of each patient, and injectable PRF was offered to them.	Total patients: 3	Following four sessions of injectable PRF therapy, two patients were content with the extent of hair growth and declined additional sessions. On the other hand, the remaining patients, including one who was particularly challenging to treat, experienced moderate hair growth, and were open to continuing with more injectable PRF sessions.
Majewska L. 2023 [22]	Retrospective study	The clinic's patients (women, ages 32–45) were assigned for observation.	PRF was prepared for injection using the whole blood of each patient and injected intradermally in the periorbital area. The patients underwent four procedures in 1-month intervals.	Total patients: 10. Age- 32-45 years	Injectable-PRF (i-PRF) injection appears to be a cost-effective, safe, and well-tolerated technique that effectively improves skin condition and produces aesthetic correction of facial wrinkles in the periorbital area, providing a potential solution for those seeking natural methods. Although the results of this observation are promising, larger controlled studies are needed to confirm the efficacy of Platelet-Rich Fibrin in skin revitalization processes.

Selection criteria

The criteria for inclusion and exclusion were stringently set to refine the quality of the studies reviewed. Inclusion required studies to involve human subjects treated with PRF for alopecia or facial rejuvenation and to report specific clinical outcomes. Excluded were non-English publications, conference abstracts, letters, reviews, expert opinions, and studies lacking detailed data on PRF preparation or application methods.

Data extraction and quality assessment

Two independent reviewers conducted data extraction using a standardized form, ensuring unbiased collection of essential information such as authorship, publication year, study design, sample size, participant demographics, PRF preparation and administration details, clinical outcomes, and main findings. The quality of the included studies was rigorously assessed using the Newcastle-Ottawa Scale (NOS) [23] for cohort and case-control studies and the NIH Quality Assessment Tool [24] for case series studies. This assessment focused on several criteria, including selection processes, comparability of study groups, the accuracy of outcome measures, and adequacy of follow-up (Table 2).

**Table 2:** The quality assessment of the included studies using the Newcastle-Ottawa Scale (NOS) for cohort and case-control studies and the NIH Quality Assessment Tool for case series studies.

Study Reference	Study Type	Assessment Tool Used	Selection (Max Points)	Comparability (Max Points)	Outcome (Max Points)	Total Score
Atsu N, et al. [17]	Prospective cohort	Newcastle-Ottawa Scale	3 (out of 4)	2 (out of 2)	3 (out of 3)	8/9
Vesala AM, et al. [11]	Original study	Newcastle-Ottawa Scale	2 (out of 4)	2 (out of 2)	2 (out of 3)	6/9
Bhoite KS, et al. [18]	Case series	NIH Quality Tool	3 (out of 4)	N/A	2 (out of 3)	5/7
Butt G, et al. [25]	Prospective study	Newcastle-Ottawa Scale	3 (out of 4)	2 (out of 2)	3 (out of 3)	8/9
Shashank B, et al. [20]	Case series	NIH Quality Tool	2 (out of 4)	N/A	2 (out of 3)	4/7
Arora R, et al. [21]	Case series	NIH Quality Tool	2 (out of 4)	N/A	1 (out of 3)	3/7
Majewska L. [22]	Retrospective study	Newcastle-Ottawa Scale	3 (out of 4)	1 (out of 2)	2 (out of 3)	6/9

Data synthesis and ethical considerations

Given the diversity of study designs and the variability in outcomes, a meta-analysis was unsuitable; a qualitative synthesis was performed instead. This approach involved categorizing the results into two main groups: effects on alopecia and effects on facial rejuvenation. It also involved summarizing the impact of PRF on clinical endpoints such as hair growth, skin texture, wrinkle reduction, and overall esthetic enhancement. The review was limited to data from previously published studies that were presumed to have adhered to ethical guidelines, negating the need for additional ethical approval for this review.

Result

The systematic review analyzed seven studies published between 2019 and 2023 involving 130 patients treated with injectable PRF. These studies were categorized based on their focus on either the treatment of alopecia or facial rejuvenation.

Alopecia Treatment Findings

Three studies, encompassing 22 patients, specifically investigated the use of PRF for treating androgenetic alopecia. The collected data demonstrated that most patients experienced noticeable hair density and growth improvements. For example, Bhoite KS et al. [18] reported that 73% of their subjects showed clinically noticeable improvements in hair growth. These outcomes suggest the efficacy and safety of PRF therapy in treating hair loss, as the treatment was well-tolerated with minimal reported side effects.

Facial Rejuvenation Findings

Four studies, including 108 patients, examined the effectiveness of PRF in facial rejuvenation. The results consistently showed improvements in skin texture and elasticity. Vesala AM et al. [11] observed moderate improvements in skin texture, color, elasticity, and the filling of soft wrinkles after three sessions of injectable PRF therapy. Similarly, in a study conducted by Butt G et al. [25], significant improvements in skin surface spots and pores were documented, with these results corroborated by patient-reported outcomes that indicated increased satisfaction with facial esthetics. Majewska L [22] further highlighted the effectiveness of PRF in the periorbital area, presenting it as a cost-effective, safe, and minimally invasive technique that improves skin condition and helps correct facial wrinkles.

Discussion

This systematic review synthesizes the current evidence on the efficacy of injectable PRF therapy in treating alopecia and facial rejuvenation. The findings from seven studies suggest that PRF can play a significant role in dermatological treatments, improving hair growth in patients with alopecia and enhancing skin quality in facial rejuvenation procedures.

Effectiveness in Alopecia

The effectiveness of PRF in treating androgenetic alopecia is supported by three key studies reviewed in this systematic analysis. Bhoite KS et al. [18], Shashank B et al. [20], and Arora R et al. [21] collectively reported favorable outcomes, with a significant proportion of patients experiencing noticeable hair density and quality improvements. These studies highlight the therapeutic potential of PRF, attributing its success to the biological actions inherent in its composition. PRF facilitates the release of growth factors such as PDGF, TGF-β, and VEGF, which are known to stimulate follicular activity and enhance angiogenesis, thereby promoting hair regrowth and increasing hair shaft thickness [26]. This mode of action is particularly effective in the scalp region, where these growth factors can directly interact with hair follicles to reverse the miniaturization process typical of androgenetic alopecia.

Facial Rejuvenation

In the realm of facial rejuvenation, four studies underscored the efficacy of PRF in improving skin quality. Atsu N et al. [17] and Vesala AM et al. [11] demonstrated significant enhancements in skin texture and elasticity and reduced fine lines and wrinkles. These studies suggest that the benefits of PRF extend beyond simple tissue repair; PRF acts as a natural filler by promoting the synthesis of collagen and facilitating tissue regeneration. The slow and sustained release of growth factors supports cell proliferation and angiogenesis and enhances the skin's structural integrity and biomechanical properties. As a result, patients treated with PRF exhibit a more youthful appearance, characterized by improved skin hydration, increased firmness, and a smoother complexion [27].

Comparison With Other Therapies

Comparative analyses, particularly the study by Atsu N et al. [17], revealed nuanced findings when PRF is juxtaposed with other regenerative therapies, such as platelet-rich plasma (PRP). While both treatments are derived from autologous blood and share some mechanisms of action, PRF has shown superior outcomes in specific esthetic parameters such as canthal smoothness and wrinkle reduction. This superiority could be attributed to the unique composition of PRF, which, unlike PRP, forms a fibrin matrix that acts as a reservoir for the gradual release of growth factors. This matrix not only prolongs the bioavailability of these crucial proteins but also ensures a sustained therapeutic effect, enhancing the long-term rejuvenation of treated areas [28]. Thus, while PRP might provide rapid results, PRF offers more enduring improvements, making it a preferable option for patients seeking lasting cosmetic benefits.

Limitations and Future Research

Despite promising findings, the studies reviewed were limited by small sample sizes, lack of control groups, and relatively short follow-up periods. These factors limit the generalizability of the results. Future studies should aim to include larger, randomized controlled trials with longer follow-ups to provide more robust data and help standardize treatment protocols. Exploring the molecular mechanisms underlying the effects of PRF could further substantiate its clinical applications [29].

## Conclusions

In conclusion, this systematic review underscores the promising potential of injectable PRF in dermatological treatment, particularly for androgenetic alopecia and facial rejuvenation. The evidence from the reviewed studies illustrates significant clinical benefits, including enhanced hair growth and improved skin esthetics, attributed to PRF's unique ability to release growth factors that stimulate natural healing and regeneration processes. Compared to other regenerative therapies, such as PRP, PRF demonstrates a sustained therapeutic effect, offering superior outcomes in specific esthetic parameters such as skin smoothness and wrinkle reduction. However, despite these encouraging findings, the current literature also highlights the necessity for larger, more rigorous clinical trials to further validate these outcomes, standardize treatment protocols, and fully exploit PRF’s therapeutic potential. Continuing research is essential to solidify PRF's standing in regenerative dermatology and optimize its application for broader clinical use.
